# Synergetic Effect of *Metschnikowia pulcherrima* and *Lachancea thermotolerans* in Acidification and Aroma Compounds in Airén Wines

**DOI:** 10.3390/foods11223734

**Published:** 2022-11-21

**Authors:** Carlos Escott, Cristian Vaquero, Iris Loira, Carmen López, Carmen González, Antonio Morata

**Affiliations:** Enotecupm, Chemistry and Food Technology Despartment, ETSIAAB, Universidad Politécnica de Madrid, Avenida Puerta de Hierro 2, 28040 Madrid, Spain

**Keywords:** lactic acid, non-*Saccharomyces*, aroma, co-inoculation, acidification, ternary fermentation, ester production

## Abstract

On the one hand, the species *Lachancea thermotolerans* is known for its high genetic diversity, allowing for the existence of strains that produce high concentrations of lactic acid. In contrast, the species *Metschnikowia pulcherrima* is renowned for its high enzymatic activity capable of producing aromatic esters during fermentation. By enhancing acidity and boosting the concentration of aromatic compounds, both species are currently used to enhance the organoleptic profile of wines. In this regard, ternary fermentations with *M. pulcherrima* and *L. thermotolerans* were carried out and the wines produced were further analysed with GC-FID, FTIR, and UV-Vis spectrophotometry. The outcomes showed that the species *M. pulcherrima* favored an increase in ethyl lactate (between 37 and 41 mg/L) along with an increased concentration of 2-phenylethyl alcohol (between 30 and 35 mg/L), whereas the species *L. thermotoleran*s was able to produce 1 g/L of lactic acid in ternary fermentations. Additionally, pH levels were slightly lower in these fermentations and the color of the white wines produced showed less chemical oxidation as hue values were lower than the control. Finally, the ternary fermentations of *L. thermotolerans* and *M. pulcherrima* had higher overall rating in the tasting. In conclusion, ternary fermentations involving these two non-*Saccharomyces* species are suggested as a substitute for spontaneous fermentations in the production of wines from neutral varieties to express freshness more vividly. This biotechnology may be further favored by the possibility of applying emerging technologies for the removal of microorganisms in grapes and musts.

## 1. Introduction

Non-*Saccharomyces* yeasts are known for providing wines with fruity, flowery, and sometimes silky features [1,2]. Such characteristics, in addition to the use of other technological approaches, such as short-term contact with lees for aroma quality improvement [3], may improve the quality of wines made from non-aromatic grape varieties, such as the widely grown *Vitis vinifera* L. Airén in Spain. Currently, the use of non-*Saccharomyces* strains in mixed or sequential inoculations is a trend in wine research due to their potential use in winemaking at the industrial level. The species commonly studied include those from the following genera: *Hanseniaspora*, *Lachancea*, and *Torulaspora*. *Hanseniaspora* spp. are usually related to both positive and negative aromatic parameters that could affect, in either way, the fermentations with strains of this species commonly found in the grape pruina as native microflora. Among negative factors is the production of biogenic amines, acetoin, and off-odors, while an increase in esters and higher alcohols may improve the aromatic profile of wines [4]. From this genus, the species *H. vineae* stands out among the other species from this genus due to a significant increase in glycerol, acetyl, and ethyl ester compounds, as well as relative decreases in alcohols and fatty acids, and sequential inoculations with *S. cerevisiae* could increase the fruitiness of wines [5]. *H vineae* belongs taxonomically to a group different from the majority of *Hanseniaspora* spp. Strains are characterized by having enzymatic activity that increases the concentration of acetate esters, such as 2-phenylethyl acetate and ethyl acetate, which increase the fruity aromas [6]. Nonetheless, most of the species of this genus are not often desired in fermentations due to its potentially high production of volatile acidity; such is the case of strains of *H. uvarum* and *H. guilliermondii* [4]. The species *Lachancea thermotolerans*, formerly known as *Kluyveromyces thermotolerans*, is able to metabolize sugars towards lactic acid production in concentrations that may reach even more than 12 g/L [7]. This metabolic feature is important from an organoleptic standpoint not only for the increase in freshness that it may produce in wines [8] but also from a quality standpoint; this is because the increase in lactic acid concentration would stabilize the wine microbiologically against spoilage, allowing a reduction of metabisulphite as an antimicrobial additive. The latter genus, *Torulaspora* spp., is known for its ability to thrive in must fermentation without previous native flora reduction and for its usage in winemaking after the well-known and widely used *S. cerevisiae* [9]. The behavior of several strains of this species has contradictory results, with some claiming that this species is capable of reducing ethanol content without the production of undesirable compounds [10], as well as the production of 3-ethoxy-1-propanol, an ethoxy-alcohol derivative from the group of dialkyl ethers with an ether-like aroma, and **γ**-ethoxy-butyrolactone with a nutty aroma [11], both of which could contribute to wines with a less expressive fruity profile or a pleasant creamy aroma, respectively.

The use of *Metschnikowia* spp., as it happens with other non-*Saccharomyces* yeasts, is uncertain not only for the high volatile acidity often associated with its metabolism but also for the production of pulcherriminic acid and further iron-complex pulcherrimin [12,13]. Pulcherrimin may slow down the implantation of other selected strains in mixed fermentation, or it may delay the thriving of native or inoculated *Saccharomyces* and non-*Saccharomyces* strains able to deplete sugars during fermentation. Despite this disadvantage of using *M. pulcherrima* in wine production, a positive increment in ester compounds is also produced [4], especially ethyl octanoate and enzymatic activity that enhance the release of volatile thiol from their precursors [14,15].

Ternary fermentations are proposed as an alternative to spontaneous fermentations in which the metabolic features of two or more species are investigated in order to improve wine complexity and quality. To achieve the desired metabolic expression, the native population may be reduced or eliminated using emerging non-thermal technologies, such as pulsed light, allowing the selected yeast to carry out the fermentations correctly [16,17]. The outcomes of the mixed fermentation of two isolated strains of *M. pulcherrima* and one strain of *L. thermotolerans* in the biological acidification of wines are presented in this article. The metabolic expression of these fermentations, in terms of organic acids production, volatile compounds accumulation, color, and sensory analysis, are compared with other fermentative biotechnologies, such as a pure culture fermentation with *S. cerevisiae*, a sequential fermentation of *L. thermotolerans* and *S. cerevisiae*, and a ternary fermentation of *H. opuntiae*, *L. thermotolerans*, and *S. cerevisiae*.

## 2. Materials and Methods

### 2.1. Yeast Strains and Growing Media

The yeast strains used in this experimental set-up were all isolated in the Food Technology Laboratory at School of Agronomic, Food and Biosystems Engineering (Universidad Politécnica de Madrid). The species used were three non-*Saccharomyces* yeast strains (*Lachancea thermotolerans* (Lt) strain L3.1, *Hanseniaspora opuntiae* (Op) strain A56, and *Metschnikowia pulcherrima* (Mp) strain M29). A fourth strain used is the commercial strain M346, also a *Metschnikowia pulcherrima* (Mp) strain (Lallemand Bio, Madrid, Spain). For the sequential fermentation and the pure culture fermentation, the strain used belonged to the species *Saccharomyces cerevisiae* (Sc) strain 7VA. 

To reach a population of at least 8 log_10_ CFU/mL for the fermentation trials, all strains were cultivated for 24 h in liquid YEPD medium at a constant 24 °C. The liquid media for the growth of yeast was made by combining 1% yeast extract, 2% bacteriological peptone, and 2% D(+)-glucose anhydrous from Panreac Qumica in Barcelona, Spain (Laboratorios Conda, Madrid, Spain). The growing medium was autoclaved at 120 °C for 15 min.

### 2.2. Must Fermentation

The must used in this vinification assay was obtained from *Vitis vinifera* L. Airén grapes. The must was obtained after pressing the paste and separating it from the skins. The must had 223 g/L total reducing sugars with 20.5° Brix and potential alcohol 11.5 (% *v*/*v*), pH of 3.8, titratable acidity of 6 g + 0.2 g/L (as tartaric acid), 143 mg/L amino nitrogen, and 130.5 mg/L ammonia. The must was pasteurized at 85 °C for 10 min, and it was then divided into 15 brown glass ISO flasks used as fermenters. The volume of the fermenters was 250 mL, filled with 220 mL of white must with 30 mL headspace on top. 

**Figure 1:**
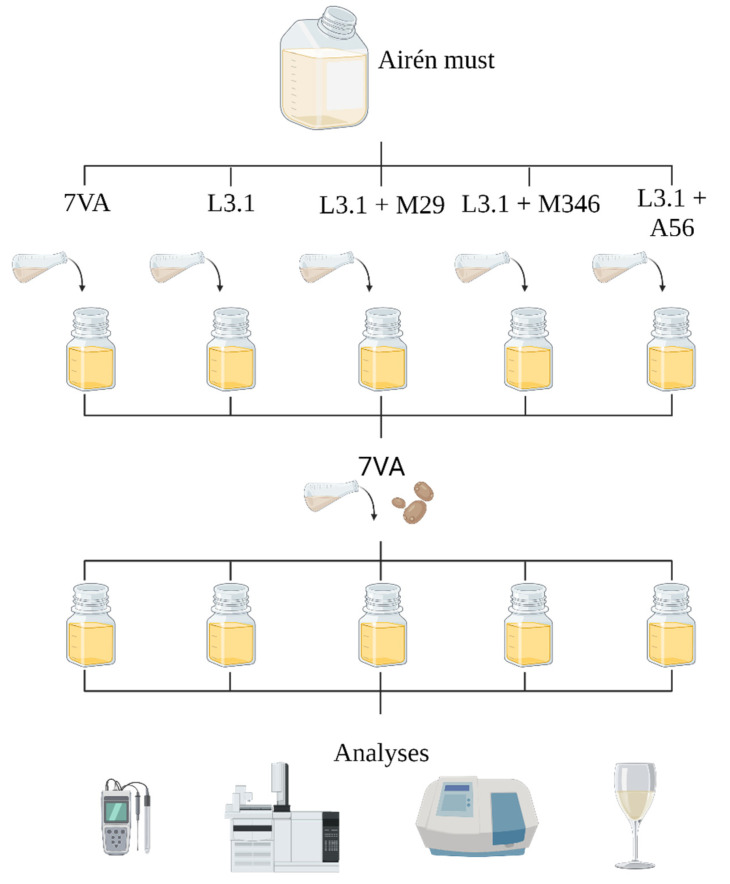
Schematic representation of the experimental set-up for the assessment of the synergetic interaction of Mp strains and L3.1.

The experiment set-up involved one control with pure culture of 7VA; a control for the biological acidification with L3.1 and 7VA in sequential fermentation; a mixed fermentation with L3.1 and A56 with sequential inoculation of 7VA; and two last mixed fermentations with each of the two Mp strains (M29 and M346) in mixed fermentation with L3.1 and the sequential inoculation of 7VA (See Figure 1). The fermenter flasks were closed with their own caps and placed at steady 19 °C inside an incubator. Each of the fermentation trial was carried out in triplicate. The production of lactic acid was followed up before the sequential inoculation of 7VA and at the end of the fermentation. The fermentations lasted over a span of 27 days.

### 2.3. General Oenological Parameters

Glucose, fructose, gluconic acid, and nitrogen compounds were followed up with a Fourier transform infrared spectroscopy (FTIR) equipment, OenoFoss™ (FOSS Iberia, Barcelona, Spain) in musts and musts under fermentation. Ethanol, glucose, fructose, and organic acids, including the volatile acidity expressed as acetic acid, were determined in finished wines. One milliliter was needed for the analysis. Ethanol was expressed as % *v*/*v*, while residual sugars and nitrogen compounds were expressed as g/L. The acids were given as tartaric acid equivalents in g/L. Lactic acid was determined with the enzymatic analyzer Y25 (Biosystems, Barcelona, Spain) through the determination of NADH produced after the reaction catalyzed with L-lactate dehydrogenase, from which L-lactic acid produces pyruvate. NADH was then read at 340 nm. Lactic acid was expressed as g/L. The pH values were measured with a GLP 21 Crison Instruments (Hach Lange Spain, S.L.U., Madrid, Spain).

### 2.4. Aroma Volatile Compounds

Volatile compounds were analyzed in accordance with a previously described method [18]. The identification of volatile compounds was carried out with an Agilent Technologies™ 6850 chromatograph (Palo Alto, CA, USA) with a column DB-624 (60 m × 250 μm × 1.4 μm). The GC-FID parameters were as follows: injector’s temperature 250 °C; detector’s temperature 300 °C; and hydrogen was the carrying gas with a flow of 2.2 L/min and split ratio of 1:10. Finally, the temperature increased from 40 °C for 5 min to 250 °C with a gradient of 10 °C/min and was maintained for 5 min. The identification and the quantification of volatile organic compounds were performed on 1 mL of previously filtered samples and the use of individual calibration curves for the following: 2-phenylethyl acetate, 2-phenylethanol, ethyl acetate, isobutyl acetate, ethyl butyrate, isoamyl acetate, acetaldehyde, methanol, 1-propanol, diacetyl, 1-butanol, 2-butanol, isobutanol, acetoin, 2-methyl-1-butanol, 3-methyl-1-butanol, ethyl lactate, 2,3-butanediol, and 1-hexanol. Lastly, in accordance with the procedure previously described, 100 μL of 4-methyl-2-pentanol (500 mg/L) was used as internal standard.

### 2.5. Color Assessment

The color representation obtained with the DNA Phone Smart Analysis—Wine (Parma, Italy) is given in CIELab coordinates which assess the luminosity or lightness (L), the green–red (a), and the blue–yellow (b) components and the CIELChuv cylindrical coordinates that provide values for chroma (C) and hue (h). The samples are placed in 0.1 cm path-length polymethyl methacrylate (PMMA) cuvettes. Samples were filtered with 0.45 μm methyl-cellulose membranes prior to the analysis. Additionally, the total polyphenol index (TPI) was determined with a UV-visible spectrophotometer 8453 from Agilent Technologies™ (Palo Alto, CA, USA). A discrete wavelength mode was selected to acquire the absorbance at 280 nm. Samples were filtered, diluted 100 times, and then placed in 1 cm path-length quartz cuvettes. The TPI was obtained by multiplying the absorbance by the dilution factor used.

### 2.6. Sensory Evaluation

A panel of 10 wine-tasting experts performed a sensory analysis of the 5 wines produced in this experimental set up. The panel of experts was cited at the Chemistry and Food Technology Department of the School of Agricultural, Food and Biosystems Engineering (ETSIAAB) at Universidad Politécnica de Madrid (Spain). The wine tasting took place in a room provided with white light and constant 21 °C. The panel was integrated as follows: 4 female and 6 male adults with ages between 25 and 50. Each expert was given an evaluation sheet with 15 basic wine descriptive attributes previously agreed upon by consensus. The parameters were rated with a five-point scale from low perception (1) to high perception (5). The exception was the hue, which was rated with a different scale: pale yellow or green (1) to golden or slightly brown (5). The attributes evaluated were appearance, aroma, mouth, and an overall note. The specific attributes ranked were color intensity, hue, transparency, intensity of the aroma, quality of the aroma, flowers, herbs, fruitiness, reduction, oxidation, acidity, astringency, body, and bitterness. The results were analyzed using a one-way ANOVA, and the significant differences are shown in the radar chart obtained. A valid informed consent for the sensory analysis was received from the panel of experts. The UPM Ethics Committee has accepted this essay in accordance with the statement AIDLAPPMLB-AMB-HUMANOS-20221026. 

### 2.7. Statistical Analysis

All samples were compared by their means and their standard deviations as well as through one-way ANOVA calculations with the least significant difference (LSD) test. A principal component analysis (PCA) was performed with the average concentration of lactic acid, main aroma compounds, and color. All the above determinations were calculated with PC Statgraphics v.XI software (Graphics Software Systems, Rockville, MD, USA). The significance was set at *p* < 0.05. 

## 3. Results and Discussion

### 3.1. Wine Composition

**Figure 2:**
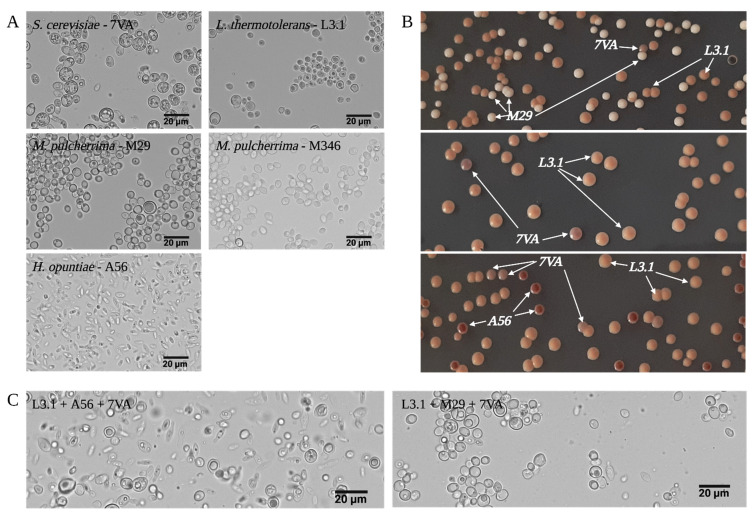
Optical microscopy images (**A**) of all used yeast strains. On the right (**B**), images of the colonies grown in CHROMAgar media to differentiate species by color. Samples got after the inoculation of 7VA and correspond to a sixfold dilution (**C**).

The micro-fermentations were completed after 28 days. The implantation of the selected yeast strains was observed by plate counts in a differential growing media with colonies in different colors (Figure 2B) since their morphology makes it difficult to be assessed in most cases by optical microscopy (Figure 2C). Only the strains A56 and Mp346 stood from the rest regarding shape and size (Figure 2A). Therefore, the use of selective growing media is useful for this purpose. The presence of Mp colonies together with L3.1 colonies, after the sequential inoculation of 7VA, suggests scarce competition between these species during fermentation. Despite the production of lactic acid in values similar to the L3.1 control wine, an interspecies synergetic growing effect with no inhibition for one another was observed in the fermentations. Regarding this and other oenological parameters discussed hereafter, there is no evidence of inhibition on L3.1 populations. Similar observations have been made in assays using other musts with different fermentative conditions (pH values, temperature of fermentation, addition of SO_2_, nitrogen content, etc.) [19]. The major risk on ternary fermentations such as these, is the antifungal effect that pulcherriminic acid may have had on the implantation and growth of L3.1 since this acid depletes iron (III) in the form of a ferric complex known to inhibit other species’ proliferation. The inhibition effect has been reported against yeast strains from spoiling species from the genera *Hanseniaspora*, *Pichia*, and *Brettanomices/Dekkera* [13]. Fermentation delays caused by *Metschnikowia pulcherrima*’s antimicrobial activity are common [4]. This ferric complex, known as pulcherrimin, is a microbial pigment, and the presence of a red halo around the colonies grown in YEPD agar media confirmed its production by the Mp strains under investigation. (Image not shown).

**Figure 3:**
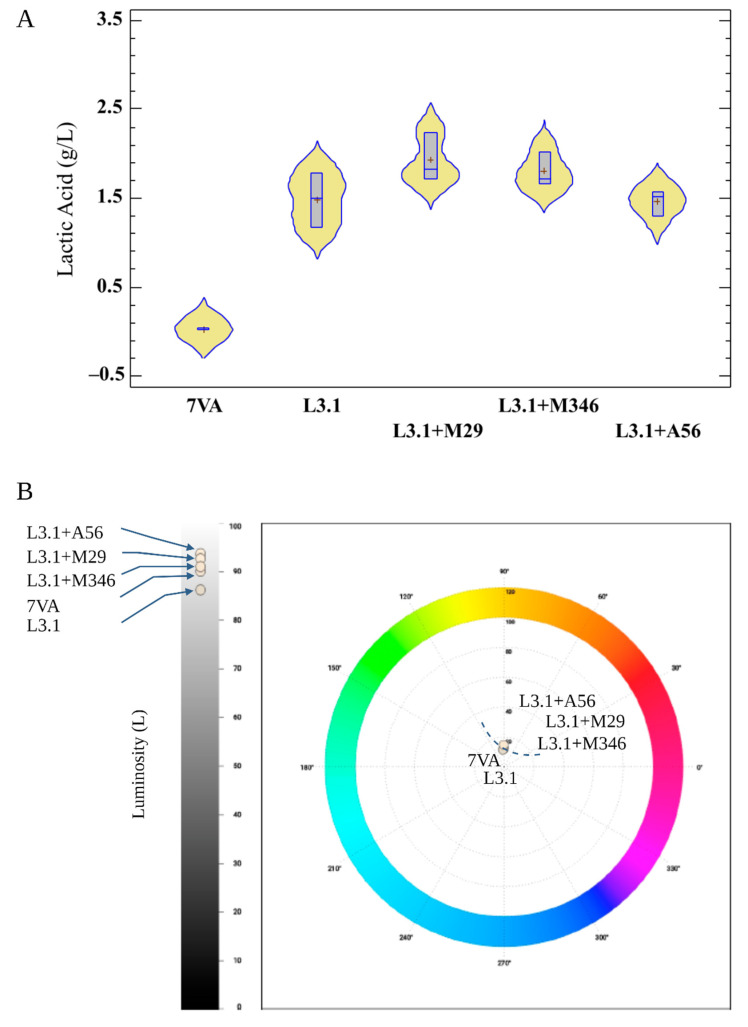
(**A**) Violin chart for the accumulation of lactic acid (g/L) in the different fermentations. Different letters indicate significant difference between means (*p* < 0.05). (**B**) Comparison of wines in terms of chromatic parameters and luminosity.

In terms of oenological parameters, all wines had less than 4 g/L sugars and similar ethanol concentration (% *v*/*v*) (See Table 1). The concentration of lactic acid was not high enough to alter the final volume of ethanol found in the fermentations (Figure 3A). Concentrations of lactic acid larger than 2 g/L usually have an impact in the volume of ethanol measured in wines [7] as well as the partial consumption of malate during fermentation. These traits are strain-related and could be associated with genetic variations observed in strains from different geographical origins and may lead to higher total acidity in wines [20]. The production of lactic acid by Lt species is strain-related, and it is also affected by other factors such as nitrogen, temperature, and SO_2_ dissolved in musts [21]. In cases such as this, where the must had barley 270 mg/L of ammonium salt and amino nitrogen together, the production of lactic acid has not been extreme, as in other cases previously reported. This is the reason why neither ethanol, nor the pH values, have differed greatly among the four fermentations where L3.1 was used (See Table 1). Nonetheless, there are statistically significant differences between the control and all the other fermentations in terms of total acidity, pH, and lactic acid, as no acidifying strains were used in the control. Finally, all fermentations resulted in similar glycerol accumulation and relatively low volatile acidity production. As a result, the metabolic performance of L3.1 used in this essay yielded close to media values when compared with high glycerol producers with concentrations of up to 8 g/L. [7]. In terms of acetic acid production, the accumulation of this organic acid in the ternary fermentations was lower than that observed in sequential fermentations with 7VA (ca. 0.3 g/L in the first case vs 0.4 and 0.6 g/L in the second) [22]. According to these analytical findings, there is no reason for consumers to perceive these wines negatively on a sensory level.

### 3.2. Aromatic Profile 

The differences in oenological parameters that have been observed do not seem to be sufficient to clearly distinguish wines from one another. Neither a significant rise in lactic acid buildup nor a slight decline in pH or alcohol volume are present. Through a sensory experience, the changes are then prone to be detected in the mouth and in the aroma profile.

The aroma compounds produced through the microbial metabolism during fermentations are summarized by type of compound in Table 1. A more detailed table with all single aroma compounds determined with GC-FID is available in Table S1. 

The fermentations carried out with M29 and M346 produced more ethyl lactate. Even though there was no statistically significant difference in the lactic acid among the fermentations with Lt, the two fermentations with Mp strains in co-inoculation with L3.1 produced ethyl lactate with substantial variances (between 37 and 41 g/L vs. 21 g/L for pure L3.1 and 7.5 g/L for A56 + L3.1—See Table S1). Similar results were seen in Merlot wine fermentations with several Lt strains, with up to 16 times more ethyl lactate produced [23]. The only common trend in ester production when using L3.1 was an increase in ethyl lactate and ethyl hexanoate, as well as a decrease in phenylethyl acetate [24]. These volatiles are responsible for the buttery–creamy aroma, green apple and anise, or fruity scents, which were partially observed in this study. There is no prior evidence on the enhanced production of ethyl lactate by the synergetic effect of Mp and Lt. What has been observed is an increase in the production of ethyl esters, which could lead to an increase in the flavor and aroma of wines (Table 1). In this regard, *Hanseniaspora* spp. are also known for increasing certain esters and higher alcohols, but the advantage of using Mp instead of Ho may be a reduction in the potential production of biogenic amines, acetoin, or increased volatile acidity [4]. The increased production of 2-phenylethyl alcohol, whose scent is described as having notes of rose petals, makes Mp strains more attractive from an industrial perspective [25]. The production of this higher alcohol was considerably increased in this experimental setting to distinguish these fermentations from those with non-ternary fermentations or from a pure culture fermentation with 7VA. In contrast with the control with 7VA, ternary fermentations produced between 30 and 35 mg/L of 2-phenylethyl alcohol. (Table S1). The concentration obtained in ternary fermentations could scarcely be recognized in the wine matrix as there are numerous elements affecting wine scent perception. The odor perception threshold for this molecule is set as 200 mg/L in 10% (*v*/*v*) ethanol–water mixture [26], which is 5.7 times higher than the maximum concentration observed in this experiment. Overall, the results observed in terms of volatile compounds are an average of each species’ expected metabolic expression. There was a limited accumulation of lactic acid but an increase in the production of ethyl lactate; there was an increase in esters and an increase in the production of higher alcohols but no discernible microbial inhibition or competition from any Mp. Ternary fermentations improved Airén wines’ aromatic composition. Differentiations in wines due to inoculation of selected strains seem largely related to the preadaptation of the strains to certain grape varieties, especially if the strains were isolated from it [27]. Thus, differentiations may be harder to distinguish when strains are used in foreign varieties. 

### 3.3. Color Assessment

On the basis of the color analysis, it appears that biological acidification protects the color of wines from rapid evolution and non-enzymatic oxidative processes. White wines are not prone to malolactic fermentation (MLF); hence, the effect of lactic acid accumulation as a protective mechanism is hardly ever observed and investigated in this wines. However, low pH levels in white wines may not only boost SO_2_ bioactivity against spoilage yeast and bacteria but also aid in the preservation of the wines’ light color because those non-enzymatic oxidative reactions happen more slowly [28]. This chemical oxidation, also referred to as non-enzymatic oxidative reactions, includes the oxidation of phenols and browning processes involving acetaldehyde and glyoxylic acid. In this experiment, all Lt fermentations resulted in wines with lower hue values than the control, indicating a smaller yellow proportion (Table 1) and possibly less oxidative processes. Higher total polyphenol index (TPI) values in the same wines, where oxidation is also apparently lower, are likewise related to this outcome. Contrary to other organic acids found in wine that can result in microbiological alterations in beverages, such as tartaric acid, malic acid, or citric acid in lesser concentrations [29], lactic acid does not have double bonds, making it more stable than other organic acids. In this way, high lactic acid concentrations reduce pH values to enhance the bio-protective effect against spoilage and the oxidative reactions. White wines with high lactic acid concentration are less prone than others to spoilage and to modifying color with ageing, even without the addition of SO_2_. In addition, some Lt strains may also have certain killer toxins against filamentous fungi [30] and other microorganisms, which may alter not only the color but also the stability of colloidal particles affecting the transparency of wines. Some additional differences observed in the wines produced with Lt are its higher luminosity and lesser chroma (Figure 3B). The luminosity or clarity is closely related to the degree of clarification and wine stability; therefore, higher values of luminosity may also reduce the possibility of having heat-unstable protein responsible for haze during wine storage as the precipitation of protein–tannin adducts happens to a higher degree at lower pH values [31]. On the other hand, chroma is expressed as color saturation whose perception may also be influenced by the lack of colloidal particles or instabilities present in the wine after filtration.

### 3.4. Variables Correlation

**Figure 4:**
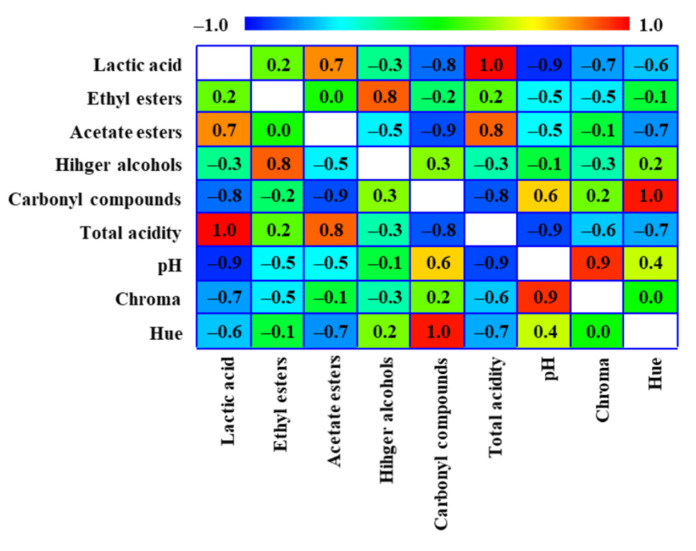
Pearson’s product-moment correlations taking into account the key variables for acidity, color, and aroma. Significance at *p* < 0.05.

There is evidence of both a strong positive and negative correlation between variables when all of the key characteristics of acidity, aroma, and color are taken into account. The coefficients between each pair of variables are accounted for in the Pearson correlations plot (Figure 4). These correlation coefficients range between −1 and +1 and measure the strength of the linear relationship between the variables. The relationships between total acidity and lactic acid, total acidity and acetate esters, higher alcohols and ethyl esters, lactic acid and acetate esters, chroma and pH, and carbonyl compounds and hue are, thus, positive and strong, indicating that both variables have the same tendency to rise or drop. However, there is a strong negative correlation between total acidity and carbonyl compounds, total acidity and pH, carbonyl compounds and acetate esters, carbonyl compounds and lactic acid, and lactic acid and pH, which can be interpreted as an increase in one variable with a decrease in the other. These correlations, as previously observed, confirm that pH decreases as lactic acid and total acidity increase, that the concentration of acetate esters increases as lactic acid accumulation increases, that carbonyl compounds are less prevalent in fermentations with higher total acidity and more lactic acid produced, and that chroma has smaller values when pH decreases. There is no association between variables for values at or near 0. In this manner, ternary fermentations with L3.1 having more lactic acid are likely to have more acetate esters, less carbonyl compounds, less chroma, and lower pH values than conventional pure culture fermentations, according to the results and the correlations study. From an analytical perspective, it is anticipated that this outcome will result in wines with a fresher profile.

### 3.5. Wine Sensory Evaluation

**Figure 5:**
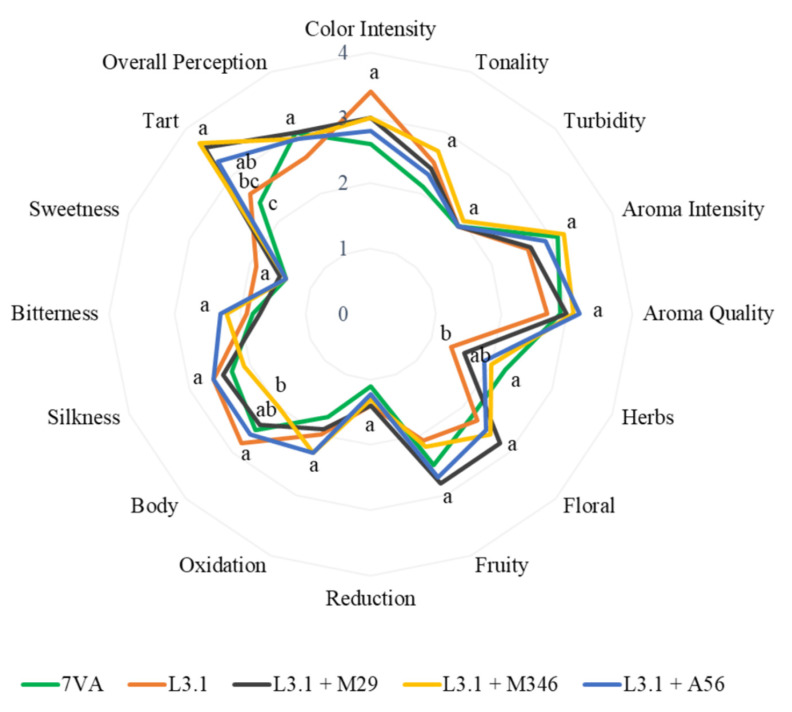
Two-dimensional radar chart for wine-tasting descriptors. Different letters indicate significant difference between means (*p* < 0.05).

The fermentative metabolites linked to the yeast strain used for fermentation produce differences in wines elaborated from a non-specific expressive grape variety such as *Vitis vinifera* L. Airén. The Airén grape variety is known for its lack of organoleptic expressivity, and it is the yeast during fermentation that can improve the aroma profile of this type of wine. In this way, the presence of fruity or floral aromas in wines is linked to metabolites produced during sugar fermentation by non-*Saccharomyces* yeasts. Yeasts from the *Metschnikowia* and *Hanseniaspora* spp. are well-known for increasing the concentration of esters in wines, but the species *Lachancea thermotolerans* is also capable of modifying the aroma profile of wines through ester synthesis and lactic acid accumulation [32]. The wines elaborated with the ternary consortium of Mp strains and L3.1 yeasts have improved the production of lactic acetate which may increase the fruity-like aroma, as it has been reported in other studies [33,34]. Despite the fact that this molecule is more abundant in ternary fermentations, statistical analysis based on sensory evaluation does not support this claim (Figure 5). These wines have higher media values because M29 and M346 fermented wines have fruitier and more floral profiles, but there are no statistical differences between these and the other wines tasted. The same ternary fermentations, on the other hand, appear to lack body as a counterpart to acid production, especially the wine fermented with M346. Tart perception is noticeably higher in all fermentations in which L3.1 was used, particularly in the ternary fermentations with Mp strains. Although the concentration of lactic acid is technically comparable to the other two wines, the perception is greater. The fact that lactic acid is present in slightly greater concentrations may increase the freshness and aroma intensity perceived in the wines. This could explain why these two wines were rated more positively in the overall evaluation. Finally, there is no significant difference between fermentations in terms of other visual and gustatory parameters.

## 4. Conclusions

The genetic diversity of non-*Saccharomyces* yeasts, and in particular, strains from the species *Lachancea thermotolerans* and *Metschnikowia pulcherrima*, is wide, yet it is for that reason that the production of lactic acid, glycerol, and other fermentative metabolites vary from one another. Ternary fermentations having *Lachancea thermotolerans* and *Metschnikowia pulcherrima* are viable to increase acidity and modify the volatile profile of wines towards fresher wines. The contribution of strains of these species affects not only their volatile composition but the color expression and the consumer perception. In order to achieve a better expression of the metabolic features of each strain, the must could be previously treated with any non-thermal emerging technology.

## Figures and Tables

**Table 1:** Chemical composition and oenological parameters of finished wines.

	**7VA**	**L3.1**	**L3.1 + M29**	**L3.1 + M346**	**L3.1 + A56**
Ethanol (% *v*/*v*)	12.6 ± 0.1^a^	9.6 ± 0.1^d^	11.9 ± 0.1^c^	12.1 ± 0.1^b^	12.1 ± 0.0^bc^
Glucose and Fructose (g/L)	1.9 ± 0.02^c^	2.0 ± 0.01^b^	2.3 ± 0.02^a^	1.5 ± 0.02^d^	2.0 ± 0.01^b^
Lactic acid (g/L)	0.0 + 0.01^b^	1.5 + 0.31^a^	1.9 + 0.28^a^	1.8 + 0.28^a^	1.5 + 0.15^a^
Acetic acid (g/L)	0.4 ± 0.01^a^	0.3 ± 0.01^b^	0.2 ± 0.00^d^	0.2 ± 0.00^e^	0.2 ± 0.01^c^
Glycerol (g/L)	5.1 ± 0.1^a^	5.1 ± 0.2^a^	5.4 ± 0.1^a^	5.1 ± 0.1^a^	5.4 ± 0.3^a^
Total Acidity ^1^ (g/L)	3.7 ± 0.0^d^	4.5 ± 0.0^b^	4.8 ± 0.1^a^	4.9 ± 0.1^a^	4.3 ± 0.0^c^
pH	3.8 ± 0.01^a^	3.6 ± 0.02^b^	3.6 ± 0.02^b^	3.6 ± 0.01^b^	3.6 ± 0.02^b^
Methanol (mg/L)	125.8 ± 3.7^ab^	114.9 ± 14.5^bc^	100.4 ± 2.3^d^	104.8 ± 3.4^cd^	129.7 ± 4.1^a^
Ethyl esters (mg/L)	1.5 ± 0.3^c^	23.0 ± 9.7^b^	42.4 ± 10.3^a^	38.7 ± 10.7^a^	9.5 ± 2.0^bc^
Acetate esters (mg/L)	18.4 ± 4.8^d^	40.7 ± 2.6^bc^	41.7 ± 5.9^ab^	33.7 ± 3.2^c^	48.2 ± 2.5^a^
Higher alcohols (mg/L)	148.2 ± 2.4^c^	230.8 ± 9.0^a^	214.1 ± 6.2^b^	218.9 ± 9.8^ab^	220.8 ± 6.1^ab^
Carbonyl compounds ^2^ (mg/L)	64.8 ± 2.5^a^	33.6 ± 6.0^c^	41.5 ± 1.5^c^	33.5 ± 2.5^b^	38.5 ± 5.9^bc^
Total Polyphenols Index	9.3 ± 0.1^d^	17.4 ± 0.1^a^	11.6 ± 0.1^c^	11.5 ± 0.1^c^	13.5 ± 0.1^b^
Colour Intensity ^3^	0.41 ± 0.00^b^	0.52 ± 0.06^a^	0.34 ± 0.00^c^	0.42 ± 0.00^b^	0.30 ± 0.00^c^
Chroma ^4^	12.7 ± 0.1^a^	9.4 ± 0.1^e^	10.8 ± 0.0^b^	10.1 ± 0.0^d^	10.4 ± 0.0^c^
Hue ^4^ (º)	86.5 ± 0.01^a^	85.4 ± 0.02^b^	83.1 ± 0.02^d^	83.1 ± 0.01^d^	83.6 ± 0.02^c^
Luminosity ^4^ (L)	90.6 ± 0.1^d^	86.8 ± 0.1^e^	93.1 ± 0.1^b^	91.5 ± 0.1^c^	94.1 ± 0.1^a^

^1^ Expressed as tartaric acid; ^2^ Comprises: acetoin, diacetyl, acetaldehyde; does not include butanediol; ^3^ Obtained from the sum of the following wavelengths: λ 420 nm, λ 520 nm, and λ 620 nm; ^4^ From CIELab and CIECh coordinates; average and standard deviation for *n* = 3. Different letters indicate statistical differences (*p* < 0.05) between treatments of each fermentative scenario.

## Data Availability

Data is contained within the article or supplementary material.

## Supplementary Materials

The following are available online at https://www.mdpi.com/article/10.3390/foods11223734/s1, Table S1: Volatile compounds from fermentative origin.
