# Supplementary material for: Synergetic Effect of Metschnikowia pulcherrima and Lachancea thermotolerans in Acidification and Aroma Compounds in Airén Wines
Source: Foods. 2022 Nov 21;11(22):3734. doi: 10.3390/foods11223734 (PMC9689907; doi:10.3390/foods11223734)
Supplement: Supplementary file 1 [file foods-11-03734-s001.zip › foods-2011683-supplementary.pdf]

Table S1. Volatile compounds from fermentative origin measured in finished wines (mg/L). Average and standard deviation for $n = 3$. Different letters indicate statistical differences ($p < 0.05$) between treatments of each fermentative scenario.

	**7VA**	**L3.1**	**L3.1 + M29**	**L3.1 + M346**	**L3.1 + A56**
Acetaldehyde	54.9 ± 1.8^{a}	26.0 ± 5.4^{c}	33.4 ± 1.4^{b}	25.3 ± 2.0^{c}	30.5 ± 5.8^{bc}
Methanol	125.8 ± 3.7^{ab}	114.9 ± 14.5^{bc}	100.4 ± 2.3^{d}	104.8 ± 3.4^{cd}	129.7 ± 4.1^{a}
1-propanol	30.4 ± 0.6^{c}	37.8 ± 1.7^{a}	34.4 ± 0.6^{b}	35.5 ± 1.3^{b}	39.8 ± 1.3^{a}
Diacetyl	1.5 ± 0.1^{a}	1.0 ± 0.9^{a}	1.6 ± 0.2^{a}	1.5 ± 0.0^{a}	1.0 ± 0.9^{a}
Ethyl acetate	7.9 ± 4.8^{d}	30.1 ± 1.5^{b}	29.4 ± 0.8^{b}	20.9 ± 1.4^{c}	36.3 ± 1.5^{a}
2-butanol	n.d.	n.d.	n.d.	n.d.	n.d.
isobutanol	14.2 ± 0.3^{d}	21.0 ± 0.7^{b}	22.5 ± 0.4^{a}	19.8 ± 0.5^{c}	21.1 ± 0.4^{b}
1-butanol	n.d.	n.d.	n.d.	n.d.	n.d.
Acetoin	8.4 ± 1.6^{a}	6.5 ± 0.1^{b}	6.5 ± 0.1^{b}	6.8 ± 0.5^{ab}	7.0 ± 1.0^{ab}
3-methyl-1-butanol	56.4 ± 1.8^{c}	96.8 ± 4.5^{a}	88.8 ± 1.9^{b}	88.8 ± 3.6^{b}	89.9 ± 1.8^{b}
2-methyl-1-butanol	24.9 ± 1.8^{c}	40.0 ± 1.8^{a}	37.1 ± 0.2^{ab}	38.2 ± 2.5^{ab}	36.8 ± 1.4^{b}
Isobutyl acetate	2.1 ± 0.3^{ab}	0.6 ± 1.0^{b}	2.5 ± 3.0^{a}	4.1 ± 1.7^{ab}	1.9 ± 2.0^{ab}
Ethyl butyrate	1.5 ± 0.3^{a}	1.3 ± 0.0^{a}	1.3 ± 0.1^{a}	1.2 ± 0.0^{a}	2.1 ± 1.4^{a}
Ethyl lactate	n.d.	21.7 ± 9.7^{b}	41.0 ± 10.2^{a}	37.4 ± 10.6^{a}	7.5 ± 0.7^{bc}
2-3 butanediol	472.3 ± 38.2^{ab}	515.5 ± 52.0^{a}	476.1 ± 39.7^{ab}	422.8 ± 41.6^{b}	425.3 ± 35.9^{b}
Isoamyl acetate	2.0 ± 0.1^{a}	2.2 ± 0.2^{a}	3.1 ± 2.4^{a}	1.8 ± 0.2^{a}	2.1 ± 0.2^{a}
hexanol	3.6 ± 0.0^{b}	4.1 ± 0.6^{ab}	4.0 ± 0.3^{ab}	3.8 ± 0.2^{b}	4.7 ± 0.6^{a}
2-phenylethyl alcohol	19.7 ± 0.4^{b}	35.2 ± 1.0^{a}	31.3 ± 3.1^{a}	34.0 ± 2.8^{a}	33.2 ± 4.2^{a}
2-phenylethyl acetate	6.4 ± 0.1^{b}	7.9 ± 0.1^{a}	6.7 ± 0.3^{b}	6.8 ± 0.5^{b}	7.9 ± 0.3^{a}
Ethyl esters	1.5 ± 0.3^{c}	23.0 ± 9.7^{b}	42.4 ± 10.3^{a}	38.7 ± 10.7^{a}	9.5 ± 2.0^{bc}
Acetate esters	18.4 ± 4.8^{d}	40.7 ± 2.6^{bc}	41.7 ± 5.9^{ab}	33.7 ± 3.2^{c}	48.2 ± 2.5^{a}
Hihger alcohols	148.2 ± 2.4^{c}	230.8 ± 9.0^{a}	214.1 ± 6.2^{b}	218.9 ± 9.8^{ab}	220.8 ± 6.1^{ab}
Carbonyl compounds	64.8 ± 2.5^{a}	33.6 ± 6.0^{c}	41.5 ± 1.5^{c}	33.5 ± 2.5^{b}	38.5 ± 5.9^{bc}
